# Supplementary material for: Delineating the Tnt1 Insertion Landscape of the Model Legume Medicago truncatula cv. R108 at the Hi-C Resolution Using a Chromosome-Length Genome Assembly
Source: Int J Mol Sci. 2021 Apr 21;22(9):4326. doi: 10.3390/ijms22094326 (PMC8122578; doi:10.3390/ijms22094326)
Supplement: Supplementary file 1 [file ijms-22-04326-s001.zip › Supplementary - Figure S1.pdf]

International Journal of
Molecular Sciences

MDPI

Supplementary material

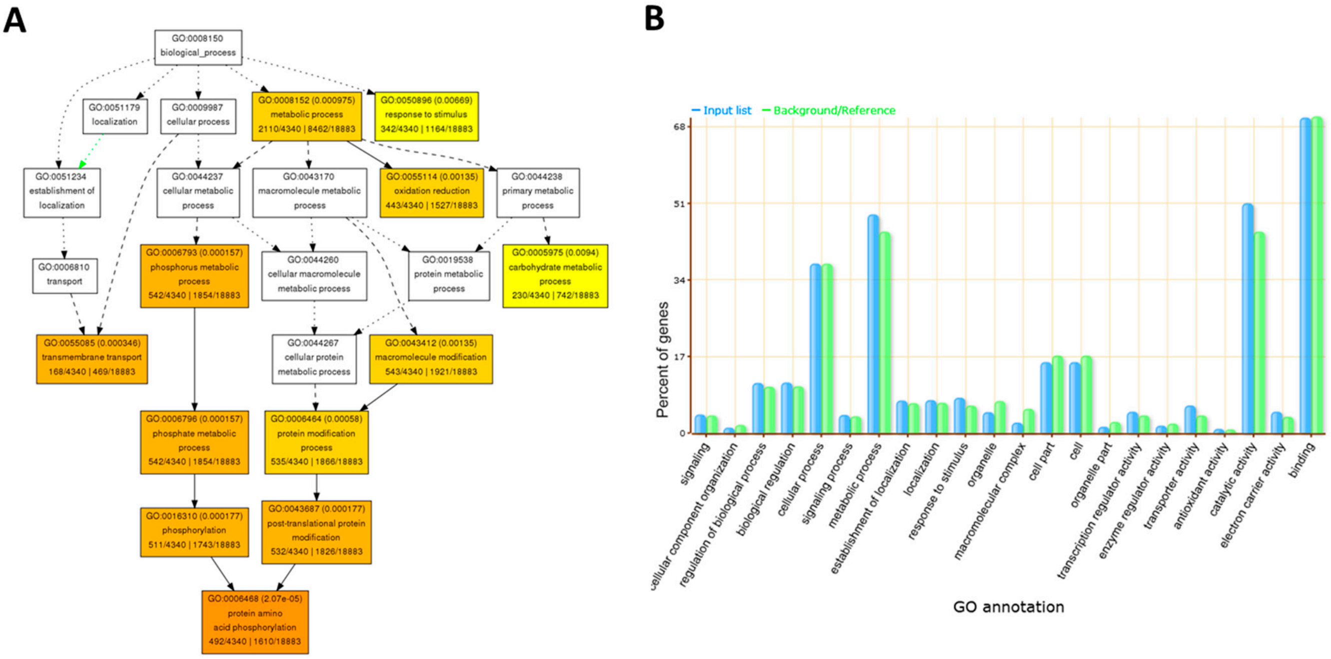

Figure S1. Pathway analysis and GO annotation of genes with *Tnt1* insertions. a) AgriGO analysis of genes specific for biological processes. Frequently inserted genes fall into the following five pathways: stress, signaling, secondary metabolism, transport, and nucleotide metabolism. b) Significant GO terms under biological processes are: response to stress, response to stimulus, defense response, protein phosphorylation, and transmembrane transport. Significant GO terms under molecular functions are: ATP binding, active transmembrane transporter activity, protein tyrosine kinase activity, and transporter activity.
